# Impact of recurrent dehydration with mild periodic water restriction on blood pressure and renal function in male spontaneously hypertensive rats

**DOI:** 10.1113/EP092812

**Published:** 2026-05-13

**Authors:** Katrina M. Mirabito Colafella, Peter N. H. Nguyen, Zoe McArdle, Debra Fong, Edmund Kwok, Kate M. Denton, Antony Vinh, Lucinda M. Hilliard Krause

**Affiliations:** ^1^ Cardiovascular Disease Program, Monash Biomedicine Discovery Institute and Department of Physiology Monash University Clayton Victoria Australia; ^2^ Centre for Cardiovascular Biology and Disease Research (CCBDR), La Trobe Institute for Molecular Science (LIMS) La Trobe University Melbourne Victoria Australia; ^3^ Department of Microbiology, Anatomy, Physiology and Pharmacology, School of Agriculture, Biomedicine and Environment La Trobe University Melbourne Victoria Australia

**Keywords:** chronic kidney disease, dehydration, hypertension, renal function, water restriction

## Abstract

The kidneys regulate fluid balance but are susceptible to adverse effects of inadequate hydration. Epidemiological studies have linked low water intake to renal dysfunction and chronic kidney disease. Previously we showed that restricting water intake to a single 2‐h period daily for 4 weeks promotes hypertension as well as renal inflammation, fibrosis and dysfunction in male spontaneously hypertensive rats (SHR). Building on these findings, the aim of the present study was to determine whether a milder, more intermittent dehydration protocol (two 1‐h periods of water access per weekday plus free access on weekends for 8 weeks) would affect renal function, renal fibrosis and inflammation, and blood pressure in the same rat model. We hypothesised that this protocol would maintain renal function, limit renal fibrosis and inflammation, and avoid further exacerbation of hypertension in male SHR. Our findings showed that the mild water restriction protocol resulted in cyclic changes in urine osmolarity, indicating periods of dehydration and rehydration. However, it did not exacerbate hypertension, accelerate renal dysfunction or increase proinflammatory processes in the kidneys. These results suggest that mild recurrent dehydration does not produce the same adverse effects observed under stricter water restriction and that the impact of recurrent dehydration on chronic kidney disease progression may depend on the severity and pattern of water restriction.

## Abstract (highlights)

- **What is the central question of this study?**
  Does recurrent dehydration with mild periodic water restriction influence kidney disease progression in male SHR?
- **What is the main finding and its importance?**
  Recurrent mild dehydration with periodic water restriction on weekdays and unlimited access on weekends for 8 weeks maintained renal function and did not further exacerbate hypertension in male SHR. These findings contrast with our previous study, where a stricter water protocol hastened the progression of kidney disease in SHR. This suggests that the degree of dehydration and regularity of rehydration periods are important factors in kidney disease progression.

## 1 INTRODUCTION

The kidneys are key regulators of fluid balance but are vulnerable to the adverse effects of inadequate hydration. Increasing epidemiological evidence suggests that chronic mild dehydration associated with suboptimal water intake may negatively affect renal health and contribute to chronic kidney disease (CKD). Exposure to repeated episodes of heat stress and dehydration has been linked to the onset and progression of kidney disease in agricultural workers (Aguilar & Madero, 2019; Crowe et al., 2022; Madero et al., 2017). Moreover, several observational studies have identified a relationship between low water intake and poor renal outcomes. Inverse associations between fluid or water intake, urine volume and progression of CKD have been reported (Clark et al., 2011; Sontrop et al., 2013; Strippoli et al., 2011; Wu et al., 2016). Moreover, high urine osmolality has been associated with a greater risk of initiating dialysis in patients with CKD (Plischke et al., 2014). Elevated serum osmolarity was also identified as an independent risk factor for the development of CKD in a large‐scale, 5‐year retrospective cohort study in Japan. Kuwabara et al. (2017) reported that a 5 mOsm/L increase in serum osmolarity carried a 24% increased risk of CKD in their study population.

Evidence also exists that a large proportion of individuals likely demonstrate poor water consumption habits. For example, Ferreira‐Pêgo et al. examined 24‐h total fluid intake in adults from 13 countries across three continents and identified the percentage of individuals complying with the European Food Safety Agency's guidelines for adequate intake of water from fluids (Ferreira‐Pego et al., 2015). The guidelines defined adequate water intake as 2.0 L per day for adult females and 2.5 L per day for adult males (European Food Safety Authority, 2010). For all the countries examined, only around 40% of men and 60% of women complied with the recommended guidelines (Ferreira‐Pego et al., 2015). In another study of 55‐ to 69‐year‐old men and women in the Netherlands, it was recorded that only a small amount of daily total fluid intake was in the form of fresh water (approximately 95 mL/day). In addition, a significant proportion of the study population, specifically 53% of men and 43% of women, indicated that they did not consume any fresh water at all (Leurs et al., 2010).

We, and others, have also shown that recurrent mild dehydration promotes kidney disease in rodents (Allen et al., 2019; Hilliard et al., 2016; Roncal Jimenez et al., 2014). We developed a water restriction model in rats that allows us to examine the effects of chronic recurrent dehydration. Rats are given access to water for 2 h per day, which induces cycles of dehydration and replenishment. Using this model, we showed that recurrent dehydration promotes significant renal pathology in male spontaneously hypertensive rats (SHR). Periodic water restriction over 4 weeks exacerbated hypertension, reduced renal function, and increased renal fibrosis and inflammatory processes (Hilliard et al., 2016).

Despite this body of evidence, gaps in our knowledge remain in terms of our understanding of how differences in patterns of water intake and regularity of rehydration periods affect kidney health and function, as well as the underlying mechanisms involved. To address this gap, we examined the effects of a milder, intermittent dehydration protocol, to be more reflective of human drinking habits, to provide further insight into the impact of irregular water intake on kidney function and morphology. Rats were allowed access to water for two 1‐h periods per weekday and unrestricted access over weekends, a schedule intended to mimic real‐world variability in hydration behaviour, as reported in human studies where beverage consumption differs by day of the week and time of day (Bellisle et al., 2010; Gibson & Shirreffs, 2013). Moreover, the experimental protocol was extended to 8 weeks to account for the longer time in which adverse effects may become apparent given the milder water protocol. We assessed the impact of this 8‐week protocol on renal function, renal fibrosis and inflammation, and blood pressure in SHR – a genetic model of essential hypertension that demonstrates a gradual decline in renal function and progression of CKD with age. Hypertensive kidney damage is typically seen in SHR from around 30 weeks of age (Hultstrom, 2012). Therefore, we investigated the impact of recurrent dehydration associated with periodic water restriction in 12‐week‐old SHR to represent the early phase of CKD. We hypothesised that this protocol would maintain renal function, limit renal fibrosis and inflammation, and avoid further exacerbation of hypertension in this model.

## 2 METHODS

### 2.1 Ethical approval

Experiments were approved by the Monash University, School of Biomedical Sciences Animal Ethics Committee (Approval number MARP1/2014/035) and were performed in accordance with the Australian Code of Practice for the Care and Use of Animals for Scientific Purposes. Moreover, we adhered to the ethical principles under which *Experimental Physiology* operates.

### 2.2 Animals

Ten‐week‐old male SHR (*n* = 21 total) were obtained from the Animal Resources Centre (Canning Vale, Western Australia, Australia). Rats were individually housed in an experimental room with temperature (maintained at 21°C) with a 12 h light/dark cycle (lights on at 07.00 h). Rats were fed a sodium‐controlled diet (0.26% w/w sodium chloride; Specialty Feeds, Glen Forrest, WA, Australia) and water ad libitum. Rats were allowed 1–2 weeks to acclimatise to these conditions prior to the commencement of the study protocol involving water restriction.

### 2.3 Baseline renal function and mean arterial pressure

At 11–12 weeks of age, rats were placed in metabolic cages for 24 h to collect a 24‐h urine sample. The urine sample was used to assess baseline renal excretory function and hydration status. Specifically, food and water consumption and urine output were recorded. Urine osmolarity (Advanced Osmometer 2020, Advanced Instruments, Needham Heights, MA, USA) and sodium concentration (EasyElectrolytes analyser, Medica, Bedford, MA, USA) were measured in the urine samples collected.

Several days later, baseline glomerular filtration rate (GFR) was assessed by measuring the clearance of fluorescein isothiocyanate (FITC)‐labelled sinistrin transcutaneously (from the skin) using a non‐invasive clearance (NIC)‐kidney fluorescent detection device (MediBeacon GmbH, Mannheim, Germany). Briefly, rats were lightly anaesthetized with 2–2.5% v/v isoflurane. The device was affixed to a depilated region on the back of the rat using a double‐sided adhesive patch and adhesive tape. Following a 3‐min baseline recording, a bolus of FITC–sinistrin (3 mg/100 g, prepared in 0.9% sodium chloride solution) was administered via the tail vein. The rat was then returned to its home cage for 2 h. At the end of the 2‐h recording period, rats were again lightly anaesthetised, and the NIC‐kidney device was removed. The collected data were analysed using NIC‐kidney device partner software which generates the elimination kinetics curve of FITC–sinistrin from which excretion half‐life (*t*_1/2_) determinations were calculated using a one‐compartment model. The half‐life was then used to calculate GFR using an empirically derived conversion factor for rats using the following formula:

$$\mathrm{GFR}\;\left(\mathrm{ml}/\min/100\;g\;\mathrm{BW}\right)=\frac{\frac{31.26\;\mathrm{ml}}{100\;g}\mathrm{BW}}{\mathrm{half}-\mathrm{life}\;\mathrm{of}\;\mathrm{FITC}\;\mathrm{sinistrin}\;\left(\min\right)}$$

(Schock‐Kusch et al., 2009).

After the completion of the metabolic cage studies and GFR measurements, each rat was anesthetized (isoflurane; 2–5% v/v O_2_) and a radiotelemetry probe (TA11PA‐C40, Data Sciences International, New Brighton, MN, USA) was implanted into the abdominal aorta for the measurement of mean arterial pressure (MAP). Specifically, a midline abdominal incision (∼5 cm) was made, and the abdominal aorta was isolated with blunt dissection. The catheter tip of the radiotelemetry probe was inserted in the abdominal aorta and secured in place with tissue adhesive (3M Vetbond, St Paul, MN, USA). The probe body was placed in the abdominal cavity above the abdominal fat and anchored to the abdominal muscles with sutures. Analgesia (carprofen; 4 mg/kg) was administered prior to surgery and for an additional 3 days after surgery. Rats were allowed to recover for 10 days in their home cage under close observation.

Following the 10‐day recovery period, basal MAP, heart rate and locomotor activity were recorded continuously for 3 days. Ten seconds of data was collected at 10‐min intervals using data acquisition software (Data Sciences International).

### 2.4 Eight‐week water treatment protocol

Rats were randomly allocated to either the control or water‐restricted treatment group, and the 8‐week treatment protocol commenced. Control SHR were given unlimited access to water on weekdays. In comparison, water‐restricted SHR were only given access to water from 09.00 to 10.00 h and from 16.00 to 17.00 h on weekdays. On weekends, both control and water‐restricted SHR had free access to water. MAP was measured continuously throughout the 8‐week treatment period. At the completion of the water restriction protocol, renal function and GFR measurements were repeated. Urinary metabolic cage studies were also performed during the water restriction protocol. GFR measurements were performed both during the water restriction protocol and immediately following the weekend when all rats had free access to water.

### 2.5 Tissue harvesting and preparation for flow cytometry

At the end of the study, rats were humanely killed by carbon dioxide asphyxiation, and a 1 mL blood sample was collected via cardiac puncture. Rats were then intracardially perfused with phosphate buffered saline (PBS; NaCl 137 mmol/L, KCl, 2.7 mmol/L, Na_2_HPO_4_ 10 mmol/L, and KH_2_PO_4_ 2 mmol/L), and the kidneys were removed. Blood samples were mixed with red blood cell (RBC) lysis buffer (NH_4_Cl 155 mmol/L, KHCO_3_ 10 mmol/L, and EDTA 0.01 mmol/L) to remove all erythrocytes and were then washed in PBS and cells were counted using a Countess Automated Cell Counter (Thermo Fisher Scientific, Waltham, MA, USA).

To isolate kidney mononuclear cells, the left kidney was enzymatically digested using collagenase type IX (125 U/mL), hyaluronidase (60 U/mL) and collagenase type I‐S (450 U/mL; all enzymes from Sigma‐Aldrich, St Louis, MO, USA) dissolved in PBS containing calcium and magnesium for 45 min at 37°C as previously described (Hilliard et al., 2016). Samples were then passed through a 70 µm cell strainer (BD Biosciences, San Jose, CA, USA) to yield a single cell suspension. After washing with PBS, cells were centrifuged at 450 *g* for 10 min at 4°C. Samples were then resuspended in 40% w/v isotonic Percoll solution (GE Healthcare, Chicago, IL, USA), and 60% w/v isotonic Percoll was gently underlaid beneath the sample for density centrifugation. Density gradients were then spun at 1385 *g* for 20 min at room temperature, with the brake off. Mononuclear cells were isolated from the interface of the Percoll layers and washed with PBS. Blood and kidney mononuclear cells were stained with an aqua live/dead viability stain (Thermo Fisher Scientific) for 15 min at 4°C. After washing with FACS buffer (PBS with 0.5% bovine serum albumin), cells were stained with fluorochrome‐conjugated antibodies (all from BioLegend, San Diego, CA, USA) for surface markers, including CD45 (leukocytes; clone OX‐1; PE‐Cy7), CD3 (T cells; clone 1F4; BV605), CD4 (T‐helper cells; clone W3/25; APC‐Cy7) and CD8 (cytotoxic T cells; clone OX‐8; PerCP). Cells were analysed using an LSR II flow cytometer (BD Biosciences) to quantitatively characterize T cell populations and tissue‐infiltrating T cells, as previously described (Hilliard et al., 2016; Vinh et al., 2010).

### 2.6 Intracellular cytokine analyses

Following preparation of mononuclear cells and digestions, cells were resuspended in complete RPMI1640 medium (fetal bovine serum, 10%; streptomycin/penicillin, 100 U/mL; HEPES, 25 mmol/L; 2‐mercaptoethanol, 2 µM) and seeded onto a 96‐well plate. Cells were then stimulated with phorbol‐12‐myristate (PMA; 50 ng/mL) and ionomycin (500 ng/mL) in the presence of Golgi transport inhibitors brefeldin A and monensin (BD Biosciences) for 6 h at 37°C with 95% O_2_ and 5% CO_2_. Following stimulation, cells were washed and stained with a viability stain and surface markers as described above. Cells were also fixed and permeabilized and stained for intracellular cytokines interferon‐γ (IFN‐γ; clone DB‐1; FITC; BioLegend), tumour necrosis factor‐α (TNF‐α; clone TN3‐19.12; PE; BioLegend), and interleukin (IL)‐4 (clone OX‐81; eFluor660; Affymetrix, Santa Clara, CA, USA). Cells were analysed using a LSR II flow cytometer (BD Biosciences). Gating for all cytokines was defined by an unstimulated blood or renal sample run concurrently with each assay. FlowJo Software Version 10.0.6 (Tree Star, Ashland, OR, USA) was used to analyse all flow cytometric data.

### 2.7 Renal fibrosis

For assessment of collagen deposition, the right kidneys were fixed in 10% neutral buffered formalin, then embedded in paraffin and sectioned at 4 µm. Sections from each kidney were stained with 0.05% w/v Picrosirius red solution and scanned at ×40 magnification using an Aperio Scanscope AT Turbo scanner (Leica Microsystems, Sydney, Australia). Forty random glomeruli and 40 non‐overlapping cortical fields without large vessels or glomeruli were then analysed from each kidney and the fraction of the stained (red) areas was determined using Aperio Image Scope software. Results are presented as percentage of glomerular or tubulointerstitial area stained with Picrosirius red.

### 2.8 Statistical analyses

The number of animals used in our study was based on power calculations (performed in GraphPad Prism, GraphPad Software, Boston, MA, USA) for the primary outcome of arterial blood pressure, using data from our previous SHR study (Hilliard et al., 2016). Assuming a population standard deviation (SD) of 7.3 mmHg, a mean difference of 14.78 mmHg, and α = 0.05, a sample size of six animals per group achieved a power of 0.8841. Data are presented as means ± SD. Daily water consumption, body weight, GFR, telemetry and metabolic cage study data were analysed using repeated‐measures analysis of variance (ANOVA), followed by Šidák's multiple comparisons test, where applicable. Student's unpaired *t*‐test (with Welch's correction) was used for group comparisons of body weight, kidney weight, arterial pressure, heart rate, locomotor activity, renal fibrosis, and circulating and renal immune cell and cytokine production data at baseline and/or at the completion of the experimental protocol. Two‐tailed *P*‐values ≤ 0.05 were considered statistically significant.

## 3 RESULTS

### 3.1 Daily water intake

**FIGURE 1:**
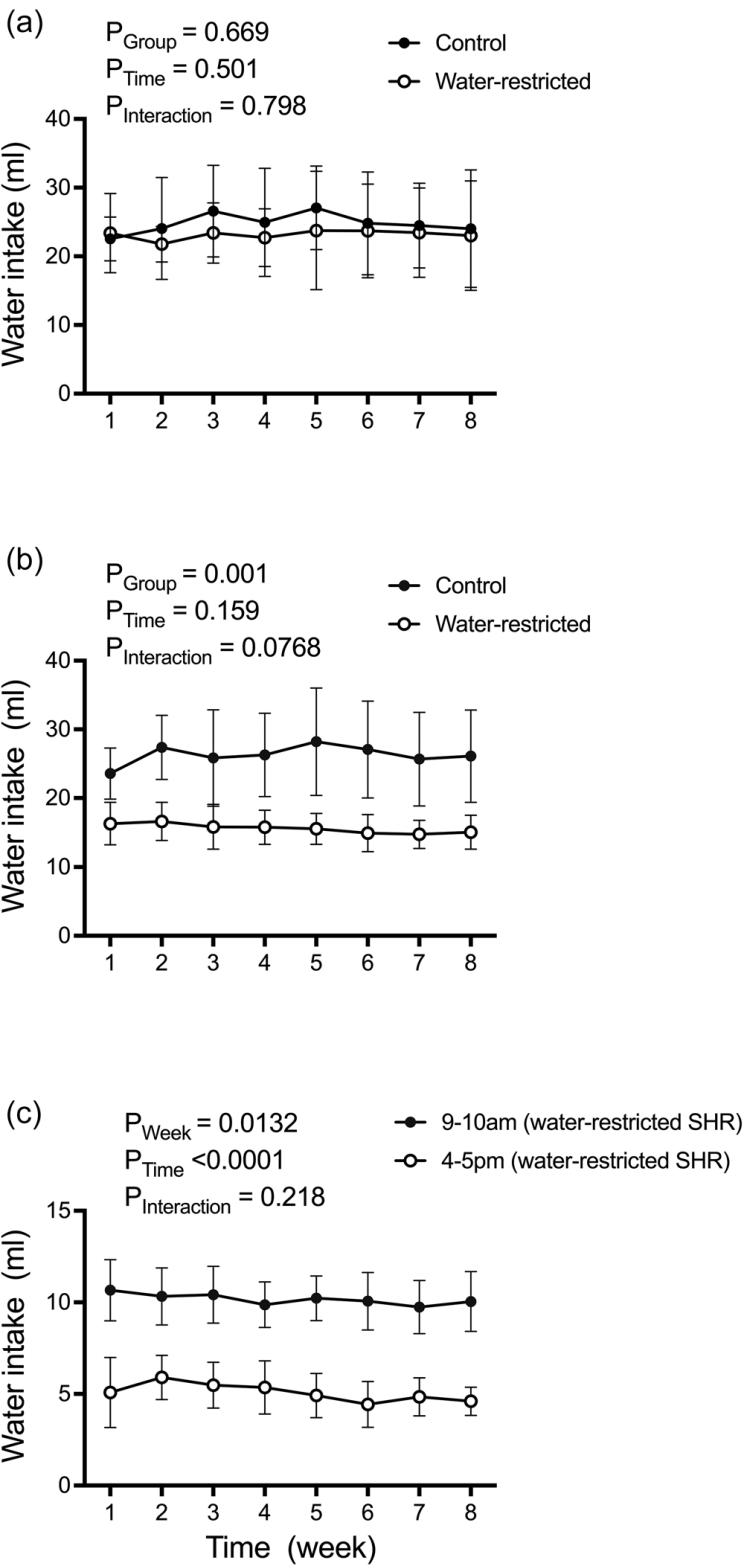
(a, b) Average daily water intake for control (filled circles) and water‐restricted SHR (open circles) on (a) weekends and (b) weekdays across the 8‐week water restriction study. (c) Average water intake for water‐restricted SHR on water‐restriction days between 09.00 and 10.00 h (filled circles) and 16.00 and 17.00 h (open circles). All data are presented as means ± SD. Data were analysed using repeated‐measures ANOVA. *n* = 6–7 per group.

Water intake was similar between the treatment groups on weekends when both control and water‐restricted SHR had free access to water (Figure 1a). During the 8‐week water restriction protocol, weekday water consumption was ∼41% less in the water‐restricted SHR than the control SHR on weekdays (Figure 1b). Moreover, on average water‐restricted SHR consumed ∼50% more water during the 09.00–10.00 h drinking period as compared to the 16.00–17.00 h drinking period on weekdays (Figure 1c).

### 3.2 Body and kidney weight

**FIGURE 2:**
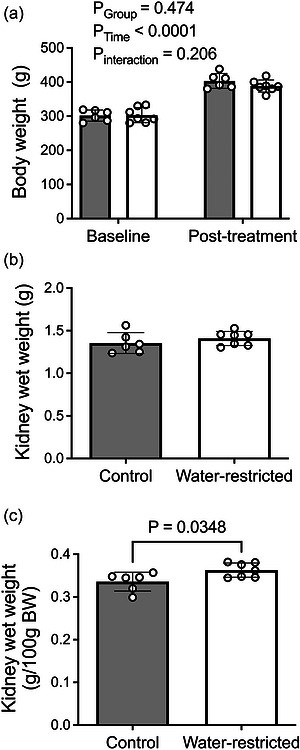
(a) Body weight in control (filled bars) and water‐restricted (open bars) SHR at baseline and at the end of the 8‐week water‐restriction protocol (post‐treatment). (b, c) Kidney weight expressed in g and g per 100 g body weight in control and water‐restricted SHR at the conclusion of the 8‐week water restriction protocol. All data are presented as means ± SD. Body weight data were analysed using repeated‐measures ANOVA followed by Šidák's multiple comparisons test (2 comparisons per analysis). Kidney weight data were analysed using an unpaired *t*‐test. #*P *< 0.05 versus control SHR. *n* = 6–7 per group.

Body weight was similar between the control and water‐restricted SHR at baseline (Figure 2a). Over time, body weight increased on average by 25% in control and 22% in water‐restricted SHR. At the end of the water restriction there was no significant difference in body weight between the treatment groups (Figure 2a).

At the completion of the water restriction protocol, average kidney weight was not significantly different between the control and water‐restricted SHR (Figure 2b). However, kidney to body weight ratio was greater in the water‐restricted than control SHR (Figure 2c).

### 3.3 Mean arterial pressure, heart rate, and locomotor activity

**FIGURE 3:**
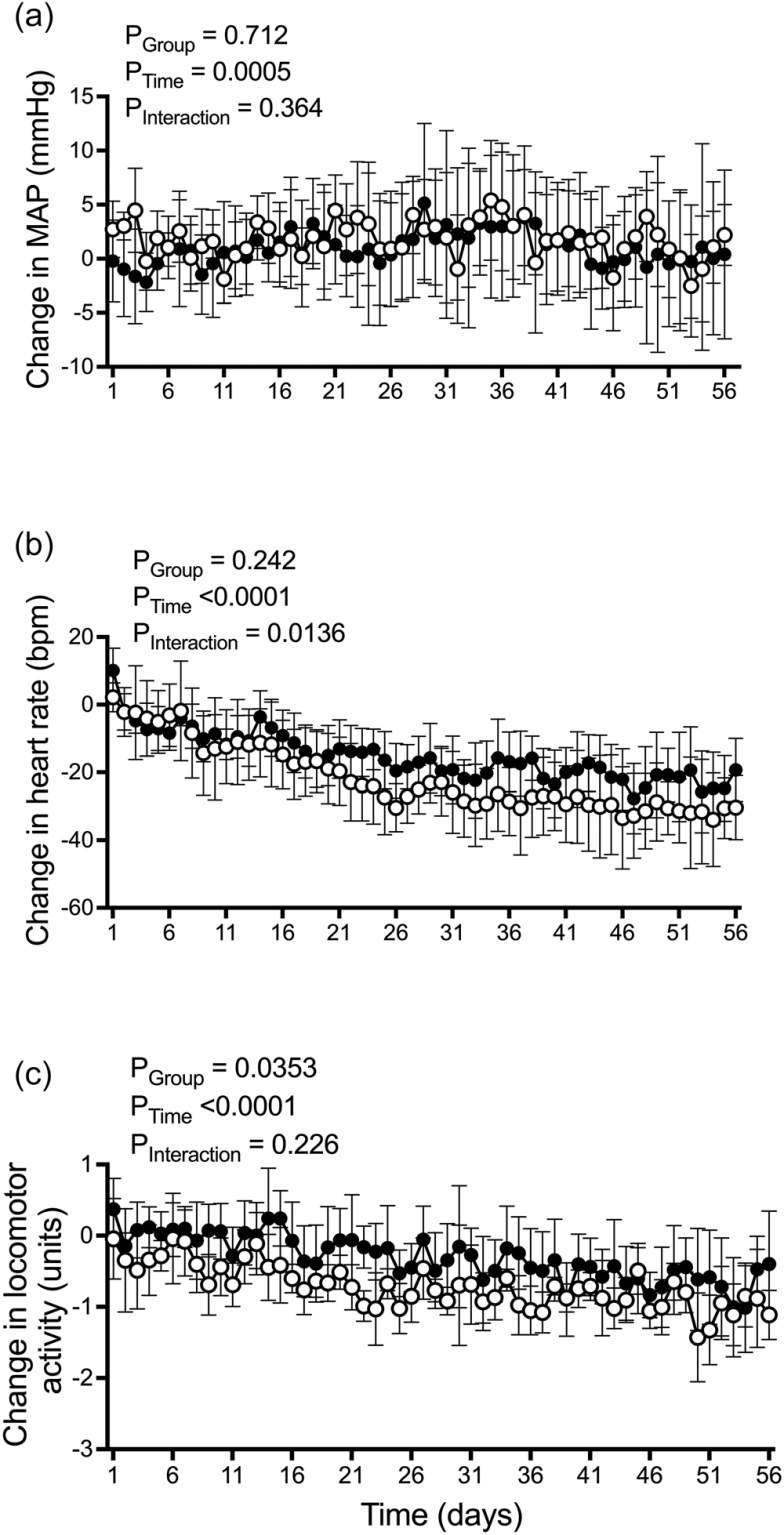
Delta change in 24‐h average mean arterial pressure (MAP) (a), heart rate (b) and locomotor activity (c) from baseline across the 8‐week protocol of water restriction in control (filled circles) and water‐restricted (open circles) SHR. All data are presented as means ± SD. Data were analysed using repeated‐measures ANOVA followed by Šidák's multiple comparisons test (56 comparisons). *n* = 6 per group.

**FIGURE 4:**
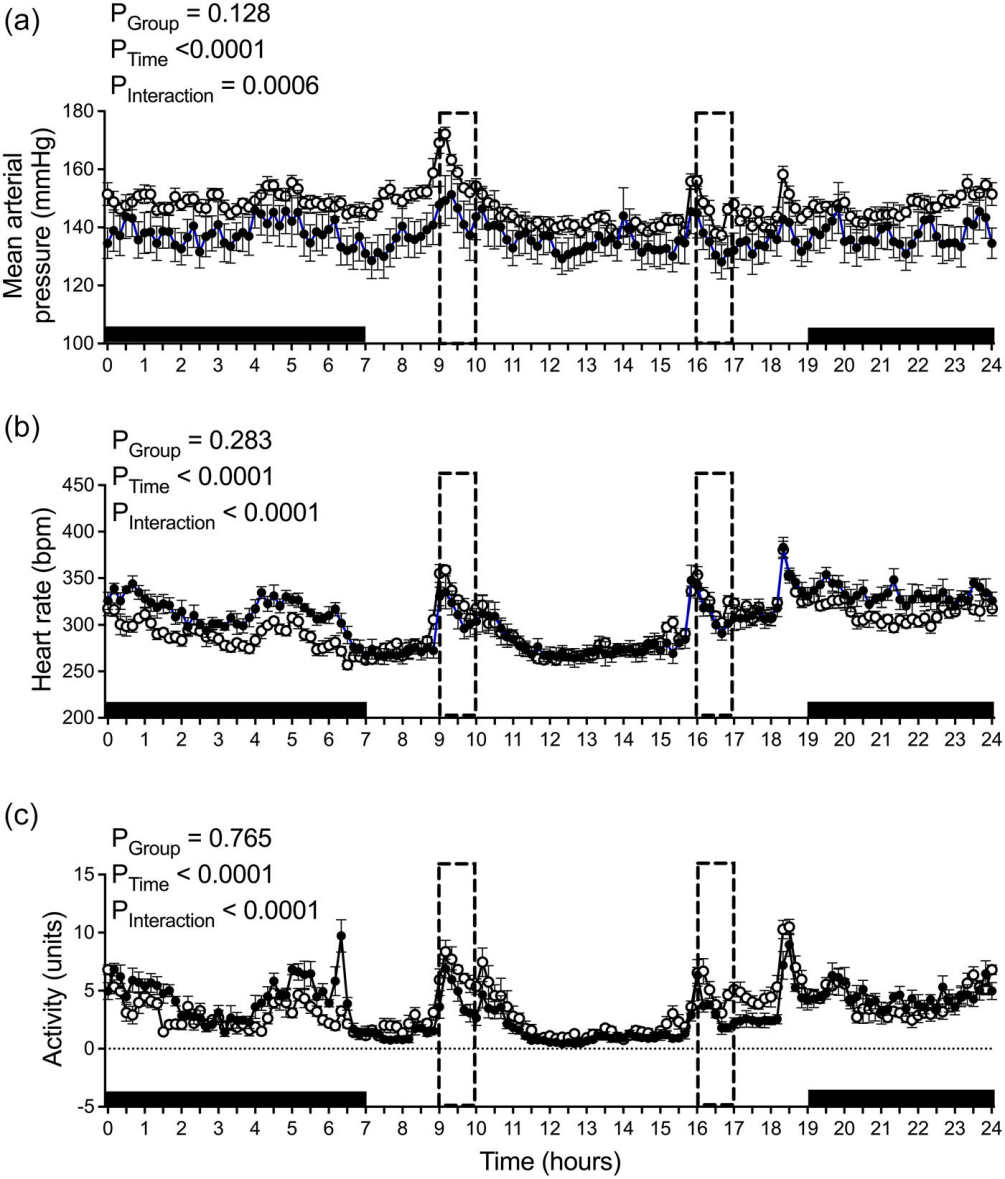
Average hourly mean arterial pressure (MAP) (a), heart rate (b) and locomotor activity (c) on weekdays during the final 2 weeks of the experimental protocol in control (filled circles) and water‐restricted (open circles) SHR. All data are presented as means ± SD. Data were analysed using repeated‐measures ANOVA. *n* = 6 per group. The dashed rectangles represent when the water‐restricted rats had free access to water.

**FIGURE 5:**
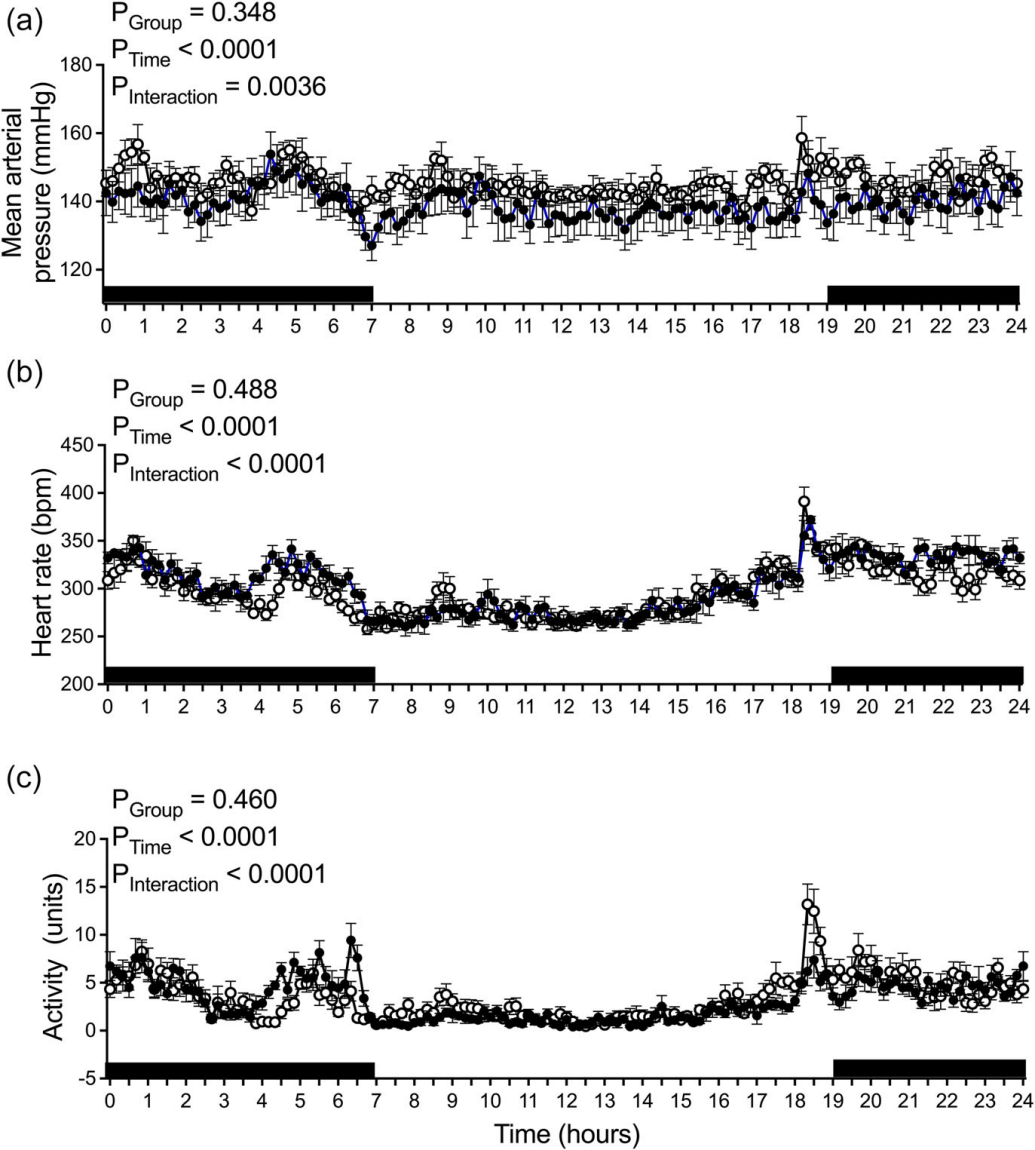
Average hourly mean arterial pressure (MAP) (a), heart rate (b) and locomotor activity (c) on weekends during the final 2 weeks of the experimental protocol in control (filled circles) and water‐restricted (open circles) SHR. All data are presented as means ± SD. Data were analysed using repeated‐measures ANOVA. *n* = 6 per group.

At baseline, MAP was similar between the control and water‐restricted SHR (139 ± 11 vs. 145 ± 5 mmHg, respectively). We did not observe any significant effect of the 8‐week water restriction protocol on MAP. Across the duration of the study, MAP remained close to baseline in both the control and water‐restricted SHR (Figure 3a). Furthermore, the circadian pattern of MAP was not significantly affected by water restriction. The day–night difference in MAP was 4 ± 2  and 5 ± 1 mmHg in the control and water‐restricted SHR, respectively, at baseline. Moreover, the average day–night difference in MAP during the final 2 weeks of the experimental protocol was 2 ± 2  and 2 ± 1 mmHg on weekdays and 4 ± 3 and 2 ± 1 mmHg on weekends in control and water‐restricted SHR, respectively. Notably, examination of hourly MAP averages across the final 2 weeks of the water restriction protocol on both weekdays and weekends revealed sharp increases in MAP around 09.00 h and 16.00 h on weekdays in both treatment groups, which reflects the times of water presentation. These spikes in MAP were not seen on weekends when both groups had free access to water (Figures 4a and 5a). Moreover, MAP tended to be greater in water‐restricted than control SHR during the night‐time period on weekdays, but not on weekends (Figures 4a and 5a).

At baseline, heart rate was also similar between the control and water‐restricted SHR (329 ± 16 vs. 328 ± 14 bpm, respectively). Heart rate decreased in both treatment groups across the duration of the experiment (Figure 3b). At the end of the experimental protocol, mean heart rate was 21 ± 9 and 30 ± 9 bpm lower than at baseline in control and water‐restricted SHR, respectively. The decrease in heart rate over time was not significantly different between the water‐restricted and control SHR. Furthermore, examination of hourly heart rate averages across the final 2 weeks of the water restriction protocol revealed that the circadian rhythm of heart rate was different between the treatment groups on both weekdays and weekends (Figures 4b and 5b). Heart rate was lower in the latter half of the night phase on both weekdays and weekends in water‐restricted as compared to control SHR. Similar to MAP, marked increases in heart rate were also observed at the times of water presentation on weekdays in both treatment groups. These spikes in heart rate were not seen on weekends (Figures 4b and 5b).

At baseline, locomotor activity was not different between the control and water‐restricted SHR (4 ± 0.6 vs. 4 ± 0.7 units, respectively). However, across the duration of the water restriction protocol, we observed differences in locomotor activity between the treatment groups (Figure 3c). This was associated with differences in activity patterns across a 24‐h period on weekdays and weekends (Figures 4c and 5c). Water‐restricted SHR were less active than control during the latter part of the night phase on weekdays and weekends. Conversely, water‐restricted SHR were more active than control SHR during the latter part of the day phase on weekdays and weekends.

### 3.4 Twenty‐four hour metabolic cage study

**FIGURE 6:**
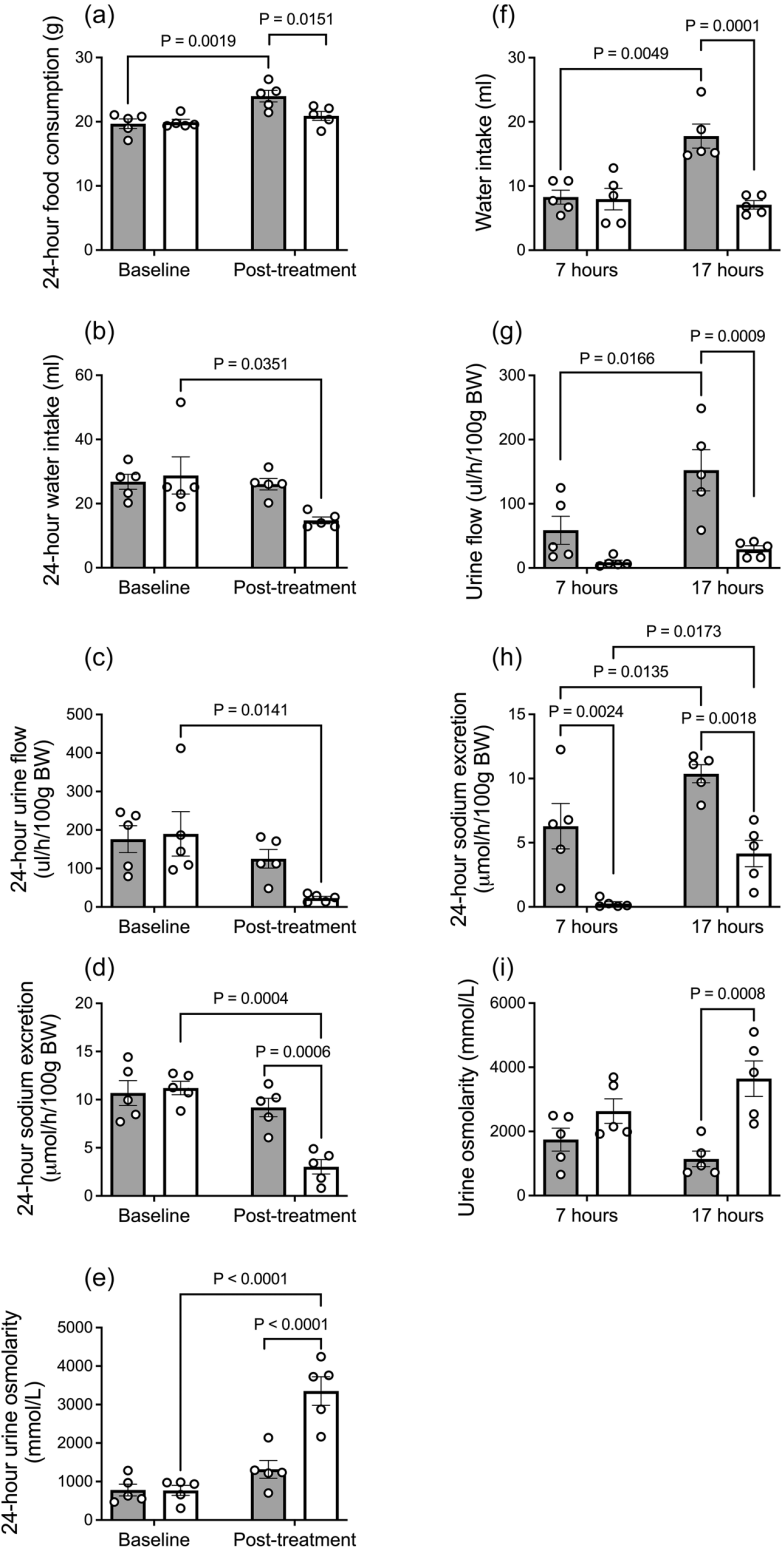
(a–e) 24‐h food consumption (a), water intake (b), urine flow (c), urinary sodium excretion (d), and urine osmolarity (e) in control (filled bars) and water‐restricted (open bars) SHR at baseline and at the end of the 8‐week water restriction protocol (on a water‐restricted day). (f–i) The 24‐h water intake, urine flow, urinary sodium excretion and osmolarity data for the post‐treatment collection period are replotted as the first 7 h and final 17 h. Data are presented as means ± SD and were analysed using repeated‐measures ANOVA followed by Šidák's multiple comparisons test (2 comparisons). *P ≤ 0.05, **P ≤ 0.01, and ***P ≤ 0.001 versus basal or 7 h. #*P* ≤ 0.05, ##*P* ≤ 0.01, and ###*P* ≤ 0.001 versus control SHR. *n* = 5 per group.

All measurements were made from 09.00 to 09.00 h (the following day) at baseline and at the end of the 8‐week protocol. Water was provided to water‐restricted rats between the hours of 09.00 and 10.00 h and 16.00 and 17.00 h. Twenty‐four hour food intake was similar in the control and water‐restricted SHR at baseline (Figures 6a). Food intake significantly increased in the control SHR across time. In comparison, there was no significant change in food intake across time in the water‐restricted SHR group (Figure 6a). At the end of the water restriction protocol, food intake was ∼13% less in the water‐restricted than control SHR (Figure 6a).

At baseline, 24‐h water intake was similar between the control and water‐restricted SHR (Figure 6b). In the control SHR, 24‐h water intake was similar to baseline at the end of the 8‐week study. Conversely, water intake was 43 ± 11% less in the water‐restricted SHR at the end of the 8‐week treatment period, as compared to baseline levels (Figure 6b). Water intake was also assessed as the first 7 h (including the 09.00 h water access period) and the final 17 h (including the 16.00 h water access period) of the metabolic cage study (Figure 6f). This analysis showed that water intake was similar between the treatment groups during the first 7 h. However, water intake was ∼60% less in the water‐restricted than control SHR during the final 17 h.

Urine flow was similar in the control and water‐restricted SHR at baseline (Figure 6c). Across time, there was no significant change in 24‐h urine flow in the control SHR. In comparison, 24‐h urine flow was 84 ± 12% less than baseline levels in rats subjected to water restriction (Figure 6c). Moreover, urine flow was ∼81% less in water‐restricted than control SHR during the final 17 h of urine collection (Figure 6g).

Urinary sodium excretion was similar between the treatment groups at baseline (Figure 6d). There was no significant change in urinary sodium excretion across time in the control SHR. Conversely, urinary sodium excretion was 73 ± 14% less than baseline levels in water‐restricted SHR at the end of the study (Figure 6d). Accordingly, at the end of the 8‐week protocol, urinary sodium excretion was ∼67% less in water‐restricted than control SHR. Furthermore, urinary sodium excretion varied across the day in both treatment groups (Figure 6h). Notably, urinary sodium excretion was significantly less in water‐restricted than control SHR during both the first 7 and final 17 h of the metabolic cage study (∼96% and 61%, respectively). Urinary sodium excretion was also greater in the final 17 h as compared to the first 7 h in both treatment groups (Figure 6h).

At baseline, 24‐h urine osmolarity was similar between the control and water‐restricted SHR (Figure 6e). In the control SHR, there was no significant change in 24‐h urine osmolarity across the 8‐week study. In contrast, 24‐h urine osmolarity was 380 ± 158% greater post‐treatment than at baseline in water‐restricted SHR (Figure 6e). Moreover, 24‐h urine osmolarity was ∼154% greater in the water‐restricted than control SHR at the end of the study. In addition, urine osmolarity varied across the day in the water‐restricted SHR. Urine osmolarity was similar in the control and water‐restricted SHR during the first 7 h of the urine collection. However, during the final 17 h of the urine collection, urine osmolarity was ∼219% greater in the water‐restricted than control SHR (Figure 6i). However, there was no significant difference in urine osmolarity between the first 7 h and final 17 h of the 24‐h metabolic cage study in either treatment group (Figure 6i).

### 3.5 Renal function and morphology

**FIGURE 7:**
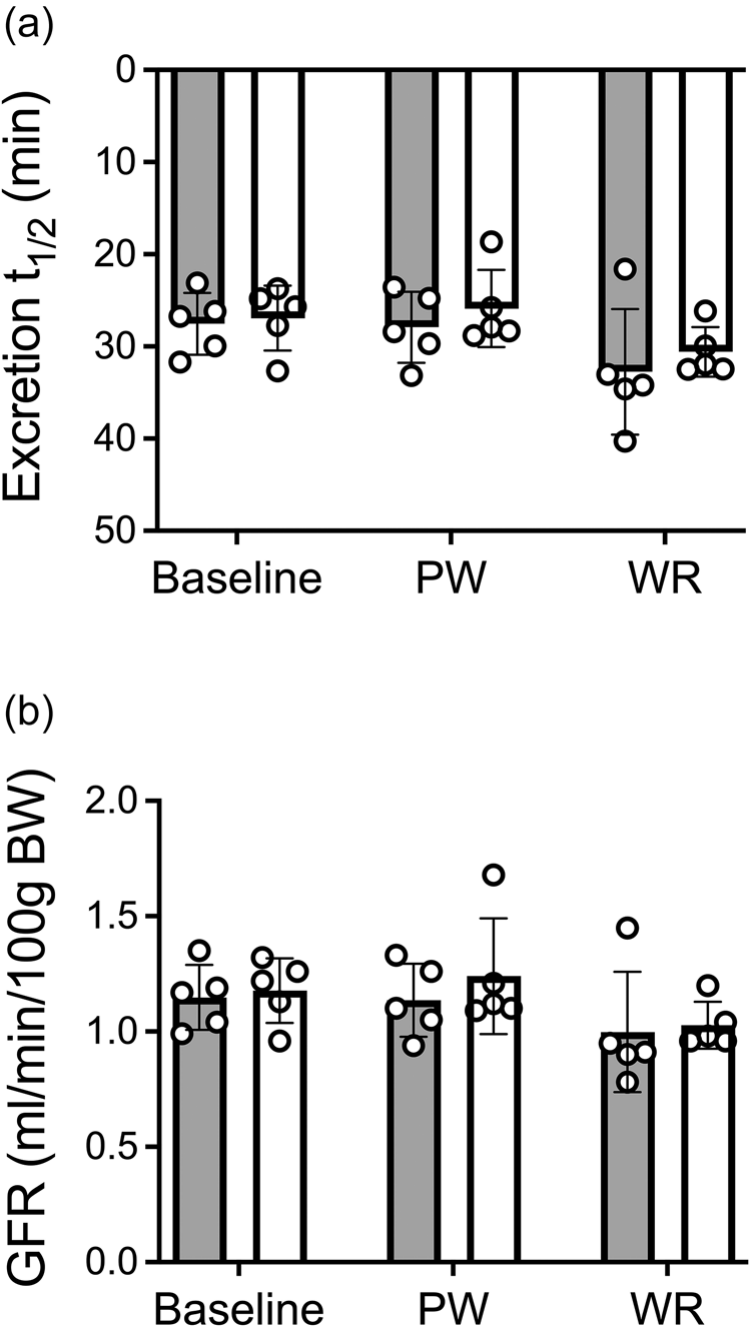
Excretion half‐life (*t*_1/2_) of FITC–sinistrin (a) and estimated glomerular filtration rate (GFR) (b) in control (filled bars) and water‐restricted (open bars) SHR at baseline and at the end of the 8‐week experimental protocol. At the end of the study measurements were obtained during the water restriction protocol (WR) and immediately post‐weekend (PW) when all rats had free access to water. Data are presented as means ± SD and were analysed using repeated‐measures ANOVA followed by Šidák's multiple comparisons test (3 comparisons). *n* = 5 per group.

Transcutaneous clearance of FITC–sinistrin and estimated GFR, corrected for body weight, were similar in the control and water‐restricted SHR at baseline (Figure 7). At the end of the 8‐week water restriction protocol, measurements of FITC–sinistrin clearance were repeated twice in all rats: immediately prior to the weekend when rats were water‐restricted, and immediately following the weekend when all rats had free access to water. There were no significant differences in FITC–sinistrin clearance or estimated GFR between the control and water‐restricted SHR, either during the water‐restriction protocol or following free access to water on weekends (Figure 7). In both treatment groups, FITC–sinistrin clearance and estimated GFR at both final time points were like that observed at baseline.

**FIGURE 8:**
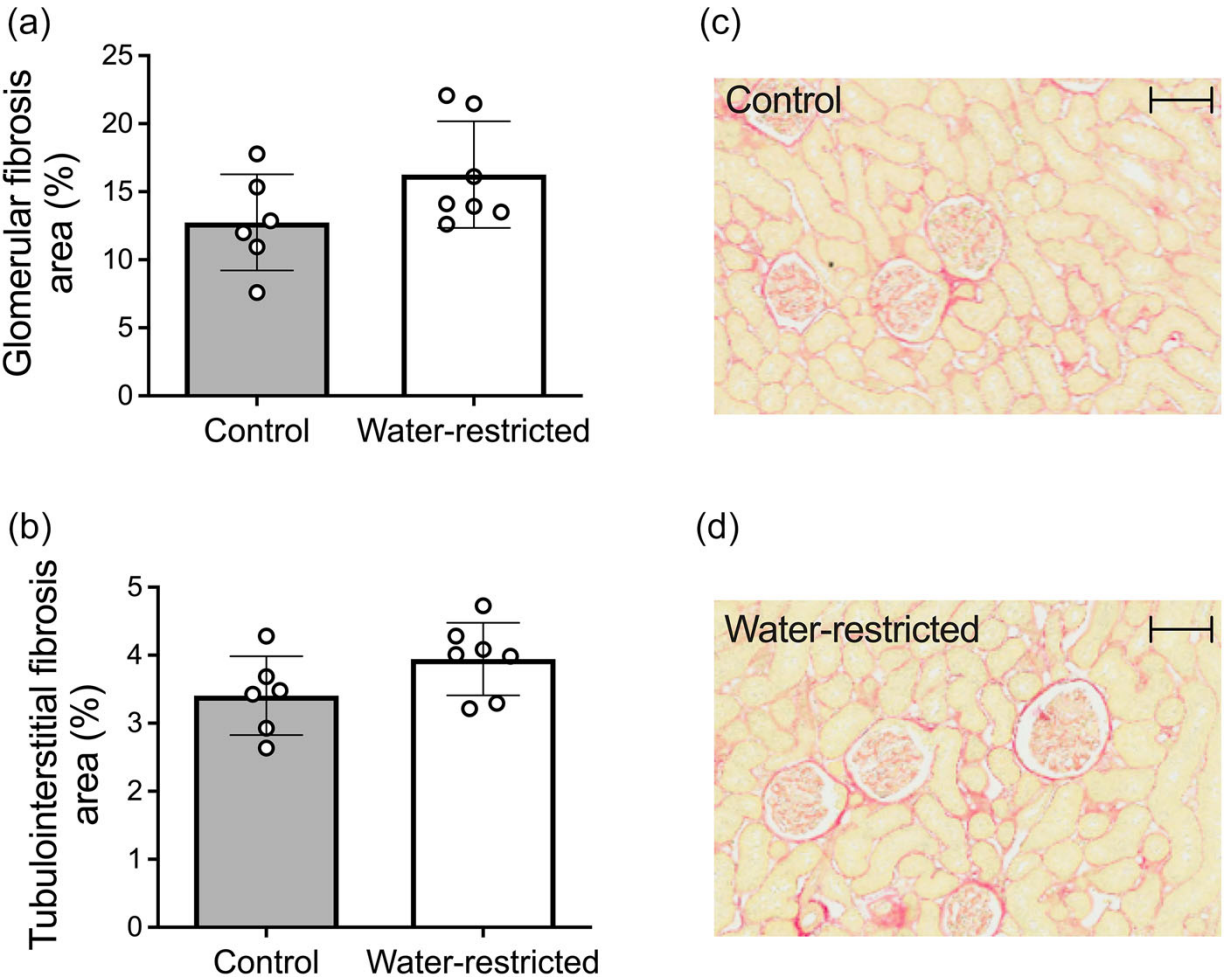
(a, b) Bar graphs represent quantification of the glomerular (a) and tubulointerstitial (b) percentage area positively stained with picrosirius red in control (filled bars) and water‐restricted (open bars) SHR. (c, d) Representative images of renal cortical fibrosis in control (c) and water‐restricted (d) SHR at the end of the 8‐week water protocol. Scale bars represent 100 µm. Data are presented as means ± SD and were analysed using an unpaired *t*‐test. *n* = 6–7 per group.

Glomerular fibrosis, as assessed by picrosirius red staining, was not significantly different between the control and water‐restricted SHR (Figure 8a; *P* = 0.12). Similarly, we did not observe any significant difference in the proportion of tubulointerstitial fibrosis between the treatment groups (Figure 8b; *P* = 0.11).

### 3.6 Immune system activation and cytokine production

**FIGURE 9:**
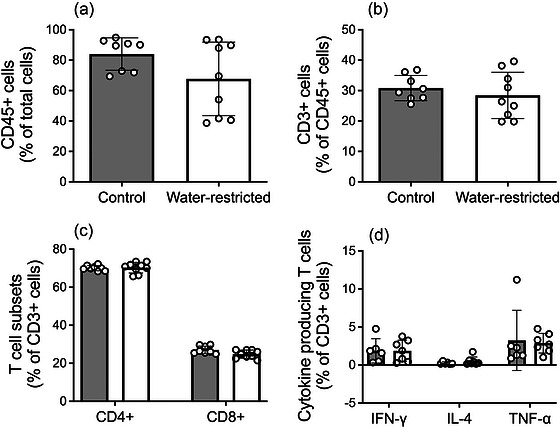
Circulating renal immune cell infiltration and cytokine production in control (closed bars) and water‐restricted (open bars) SHR at the end of the 8‐week water restriction protocol. (a) Circulating leukocytes (CD45^+^ cells), (b) T cells (CD3^+^ cells), (c) the proportion of circulating T helper cells (CD4^+^ cells) and cytotoxic T cells (CD8^+^ cells), and (d) the proportion of CD3^+^ T cells producing IFN‐γ, IL‐4 and TNF‐α. Data are presented as means ± SD. Data were analysed using an unpaired *t*‐test. *n* = 6–9 per group.

There were no significant changes in the proportion of circulating leukocyte (CD45^+^ expressed relative to live cells) count between the control and water‐restricted SHR (Figure 9a). The proportion of circulating CD3^+^ T cells and T cell subpopulations, characterized by CD4^+^ (T helper cells) and CD8^+^ (cytotoxic T cells), were also similar between the treatment groups (Figure 9b,c). Moreover, we did not observe any significant differences in the expression profiles of circulating T cell‐derived cytokines (IFN‐γ, IL‐4 and TNF‐α) between the control and water‐restricted SHR (Figure 9d).

**FIGURE 10:**
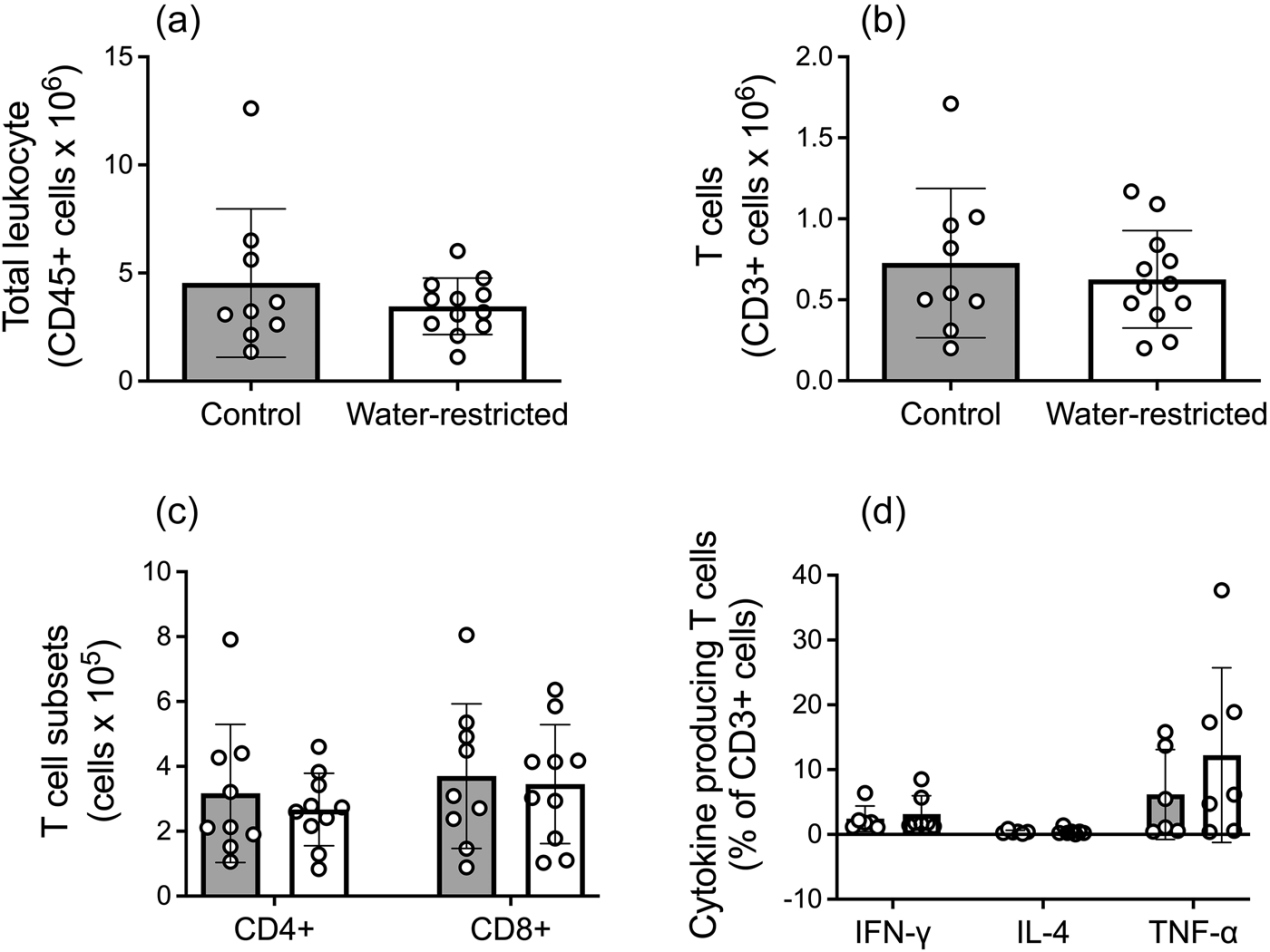
Renal immune cell infiltration and cytokine production in control (filled bars) and water‐restricted (open bars) rats at the end of the 8‐week water restriction protocol. (a) Renal leukocytes (CD45^+^ cells), (b) T cells (CD3^+^ cells), (c) the proportion of circulating T helper cells and cytotoxic T cells, and (d) the proportion of CD3^+^ T cells producing IFN‐γ, IL‐4 and TNF‐α. Data are presented as means ± SD. Data were analysed using an unpaired *t*‐test. *n* = 6–12 per group.

In the kidney, leukocyte (CD45^+^) and T cell (CD3^+^) infiltration was similar between the control and water‐restricted SHR (Figure 10a,b). Moreover, there was no significant difference in the proportion of the T cell subpopulations between the treatment groups (Figure 10c). The expression profiles of renal T‐cell derived cytokines (IFN‐γ, IL‐4, and TNF‐α) were also similar between the control and water‐restricted SHR (Figure 10d).

## 4 DISCUSSION

The major findings of the current study were that recurrent mild dehydration, associated with periodic water restriction on weekdays and unlimited access to water on weekends, for 8 weeks did not negatively impact renal health or function, or worsen hypertension in male SHR. These findings contrast with those from our previous study in male SHR, where we showed that a more severe water protocol exacerbated hypertension, reduced renal function, and increased renal fibrosis and evidence of inflammation over 4 weeks (Hilliard et al., 2016). Thus, collectively, our findings support the notion that the degree of dehydration is a salient factor in kidney disease progression. Moreover, our results suggest that the association between recurrent mild dehydration and periodic water intake and the promotion of kidney disease is likely dependent on drinking patterns and the regularity of rehydration periods.

The results of the present study extend our previous work in this field and support the importance of regular hydration periods for the maintenance of kidney health, in the face of chronic low water intake. In our previous 4‐week study in male SHR, rats were given access to water for 2 h per day (for 7 days per week) between the times of 09.00 and 11.00 h (Hilliard et al., 2016). In comparison, here we investigated the impact of a milder water restriction protocol, as induced by limiting water accessibility to male SHR to two 1‐h periods (6 h apart; 09.00–10.00 h and 16.00–17.00 h) each weekday for 8 weeks and providing rats with free access to water on weekends. Cycles of dehydration and replenishment were achieved using this milder water restriction paradigm, as evidenced by periods of high and low urine osmolarity on days in which rats were subjected to limited availability of water. On such days, total water intake was on average around 40% less in water‐restricted than control SHR, which is comparable to that observed in our original study, where rats were subjected to a more severe water restriction protocol. Notably, it was also observed that water‐restricted rats consumed most of their daily water intake (∼65%) during the water access period which closely followed the end of the dark phase (09.00–10.00 h). This was also the time point following the longest period (16 h) across the 24‐h day where rats did not have access to water. In the current study, water intake was comparable between the treatment groups on weekends, when all rats had unlimited access to water.

The water restriction paradigm adopted in the current study did not exacerbate hypertension in the male SHR, at least after 8 weeks. Moreover, we did not detect any impact of the water restriction protocol on GFR or renal morphology, as indicated by assessment of the FITC–sinistrin clearance and renal fibrosis. Importantly, assessment of FITC–sinistrin clearance and calculation of estimated GFR were made at both the end of a dehydration period and immediately following the weekend period, when all rats had free access to water, to allow us to identify any differences in renal function between the dehydrated and replenished states. In addition, we did not observe any differences in immune cell infiltration, or T‐cell derived cytokine production in the circulation, or locally in the kidney, which might serve as early markers of renal injury. These observations are in stark contrast to that of our previous study, where we observed that renal T cells polarised to a proinflammatory Th1 phenotype with an increased Th1:Th2 ratio, in a more restrictive chronic recurrent dehydration model (Hilliard et al., 2016).

Also, of interest is our observation that average kidney weight, when corrected for body weight, was greater in the water‐restricted than control SHR. This finding mirrors our previous results under severe water restriction (Hilliard et al., 2016). However, we acknowledge that absolute kidney weight did not differ significantly between control and water‐restricted groups in either study. The increased kidney‐to‐body weight ratio may therefore reflect changes in in body composition or fluid balance rather than true renal hypertrophy.

It is also possible that this ratio reflects renal adaptation to chronic alterations in water intake and urine concentration status. In this context, Bankir et al. (1988) reported a positive association between water restriction and kidney weight relative to body weight in normal Wistar rats subjected to daily water restriction for 6 weeks. They attributed this to sustained stimulation of urine concentrating processes, as supported by morphological changes in kidney zones involved in urinary concentration. Similar changes were observed under chronic treatment with the arginine vasopressin (AVP) analogue dDAVP, though the effects were noted to be more pronounced in the dDAVP‐treated rats (Bankir et al., 1988).

Notably, urinary sodium excretion was less in water‐restricted than control SHR at the end of the 4‐week study. This might be at least partly attributable to the fact that water‐restricted SHR consumed less food than their control counterparts in the current study. Moreover, it is also possible that the difference in urinary sodium excretion rates between the control and water‐restricted SHR is related to differences in circulating AVP levels. Certainly, there is evidence from both animal and human studies which supports the notion that AVP promotes antinatriuretic effects. For instance, in conscious normotensive Wistar rats, Perucca et al. (2008) reported an increase in sodium retention in response to acute administration of the vasopressin type 2 receptor agonist dDAVP. Similarly, treatment with dDAVP was shown to reduce sodium excretion in healthy human subjects, as well as subjects with nephrogenic diabetes insipidus related to mutations of *AQP2* (Bankir et al., 2005). Moreover, in other human studies it has been demonstrated that urinary sodium excretion is reduced under conditions of low urine flow and high vasopressin levels (Bouby & Fernandes, 2003).

Given our lack of evidence for a negative impact of the water restriction protocol implemented in the current study on arterial pressure and kidney function and morphology, it seems plausible that the differences observed between the current study and our previous 4‐week study in rats subjected to a more severe water restriction protocol are influenced, at least to some degree, by differences in plasma AVP levels achieved and the time period for which increased levels of plasma AVP levels are sustained. Certainly, an increase in plasma AVP was likely to be sustained over a greater time frame in the 4‐week study, given that the longest period without water intake was 22 h in our previous study, as compared to 16 h in the current study. We did not directly measure changes in plasma AVP levels, or its surrogate marker, copeptin, which we acknowledge is a limitation of this work. However, urine osmolarity was measured, which has previously been shown to reflect circulating AVP levels (Guelinckx et al., 2016).

Certainly, it has long been recognised that the antidiuretic hormone, AVP, plays a critical role in maintaining whole‐body homeostasis under conditions of water deprivation. However, accumulating evidence suggests that chronic elevation of AVP may have deleterious effects on renal health and contribute to the development and progression of kidney disease (Bankir et al., 2013). Elevated AVP levels have been reported in animal models of kidney disease (Bouby et al., 1990; Sugiura et al., 1999) and administration of the V_2_ receptor agonist desmopressin has been shown to exacerbate the development of renal injury in mice subjected to heat stress and water restriction (Roncal‐Jimenez et al., 2017). Furthermore, elevated plasma copeptin, a marker of endogenous AVP levels, has been positively associated with the development and progression of CKD in the general population (El Boustany et al., 2018) and with accelerated renal function decline in transplant recipients (Meijer et al., 2009). Accordingly, this hormone represents a likely contributor to the development of CKD under conditions of chronic recurrent dehydration.

Of course, we also cannot rule out the contribution of other mechanisms that may be differentially activated by the two dehydration protocols and contribute to kidney injury and dysfunction, such as the renin–angiotensin system or hyperuricaemia, as previously discussed (Hilliard et al., 2016). Therefore, in terms of directions for future research it would be of interest to perform additional blood and tissue analyses to provide mechanistic insight into whether differences in activation of these pathways could explain the differences in findings between the current study and our previous study in water‐restricted SHR. It would also be interesting to assess the impact of our mild water restriction protocol in SHR without ad libitum access to water during weekends (i.e., water restricted for 7 days per week). This would provide further insight into whether extended periods of water consumption, following bouts of dehydration, are protective of kidney health, and could potentially explain the lack of effect of the water restriction protocol in the current study.

The selected schedule – two discrete 1‐h access periods during weekdays followed by unlimited access on weekends – was designed to mimic fluctuations in water availability rather than continuous ad libitum access. However, this structure may provoke compensatory increases in intake, alter drinking patterns (e.g., higher rate per hour during water access periods) and thereby influence outcome variables. These factors should also be considered when interpreting the data.

A key limitation is the small sample size and limited statistical power for secondary outcomes such as renal fibrosis. *Post hoc* analysis shows that detecting the observed differences (13% for tubular and 20% for glomerular fibrosis) with 80% power at a two‐sided significance level of 0.05 would require much larger groups – approximately 21 animals per group for tubulointerstitial fibrosis and 19 per group for glomerular fibrosis. This confirms the study was underpowered for these outcomes. Although minor increases in fibrosis cannot be excluded, achieving adequate power would require sample sizes that are not feasible given ethical and logistical constraints. Future studies should include larger cohorts or longer durations to improve statistical power and clarify whether subtle changes in kidney structure or function emerge over time. Moreover, studies with larger cohorts could enhance the robustness of findings and further elucidate the relationship between hydration patterns and kidney health.

It would also be beneficial in future studies to measure additional markers of kidney function and injury, to provide a more comprehensive overview of the physiological status of the kidney under conditions of mild chronic recurrent dehydration. To that end, it is possible that mild changes in kidney structure and/or function, which might ultimately hasten the progression of CKD, developed in the rats subjected to chronic water restriction in the current study, but were not detectable using the methods employed. As discussed earlier, a large proportion of individuals likely fail to drink water regularly enough throughout the day to maintain adequate hydration. Measurements of a range of markers of kidney injury in future studies could ultimately provide insight into biomarkers that could provide a means of identification of individuals at risk of recurrent‐dehydration‐related kidney disease, and for early detection of renal pathology under these conditions.

In conclusion, our findings underscore the importance of hydration patterns – particularly the timing and regularity of water consumption – in influencing kidney health. Although severe dehydration accelerates CKD progression, our results suggest that mild, intermittent dehydration with regular rehydration may not adversely affect kidney health. Future studies are warranted to further interrogate the mechanisms underlying recurrent dehydration‐induced alterations in cardiovascular and renal function associated with chronic low water intake.

## AUTHOR CONTRIBUTIONS

Lucinda M Hilliard Krause, Katrina M Mirabito Colafella, Kate M Denton, and Antony Vinh conceived the experiments. Lucinda M Hilliard Krause, Peter N H Nguyen, Katrina M Mirabito Colafella, Debra Fong and Kate M Denton completed the water restriction studies and renal function experiments. Edmund Kwok completed the renal fibrosis analysis. Katrina M Mirabito Colafella and Antony Vinh completed the flow cytometry experiments. Lucinda M Hilliard Krause, Peter N H Nguyen, Zoe McArdle, Kate M Denton and Antony Vinh analysed the data. All authors contributed to the preparation of the manuscript. All authors approved the final version of the manuscript and agree to be accountable for all aspects of the work in ensuring that questions related to the accuracy or integrity of any part of the work are appropriately investigated and resolved; and all persons designated as authors qualify for authorship, and all those who qualify for authorship are listed.

## CONFLICT OF INTEREST

None declared.

## Data Availability

The data that support the findings of this study are available from the corresponding author upon reasonable request.
